# Mutations in Homologous Recombination Genes and Loss of Heterozygosity Status in Advanced-Stage Breast Carcinoma †

**DOI:** 10.3390/cancers15092524

**Published:** 2023-04-28

**Authors:** Brooke B. Bartow, Gene P. Siegal, Ceren Yalniz, Ahmed M. Elkhanany, Lei Huo, Qingqing Ding, Aysegul A. Sahin, Hua Guo, Cristina Magi-Galluzzi, Shuko Harada, Xiao Huang

**Affiliations:** 1 Department of Pathology, The University of Alabama at Birmingham, Birmingham, AL 35294, USA; 2 Department of Radiology, The University of Alabama at Birmingham, Birmingham, AL 35294, USA; 3 Department of Breast Medical Oncology, Division of Hematology & Oncology, The University of Alabama at Birmingham, Birmingham, AL 35233, USA; 4 Department of Pathology, Division of Pathology/Lab Medicine, The University of Texas MD Anderson Cancer Center, Houston, TX 77030, USA

**Keywords:** advanced-stage breast carcinoma, homologous recombination genes, BRCA, loss of heterozygosity, molecular profiling

## Abstract

**Simple Summary:**

The clinical significance of next-generation sequencing coupled with HRR gene analysis of the benefit of poly (adenosine diphosphate-ribose) polymerase inhibitor (PARPi) treatment in patients with breast cancer is unknown. We analyzed the tumor mutations in homologous recombination (HRR) genes and the loss of heterozygosity (LOH) score in 63 patients with advanced-stage breast carcinoma. We found an HRR gene mutation and an LOH-high score were associated with unfavorable pathological features. Comprehensive genomic profiling revealed that a subset of breast carcinomas with an HRR gene mutation other than *BRCA1/2* had a low LOH score. In order to identify potential eligible patients for PARPi therapy, appropriate testing is warranted and requires further investigation.

**Abstract:**

Poly (adenosine diphosphate-ribose) polymerase inhibitors (PARPis) have demonstrated antitumor activity in cancers with a homologous recombination deficiency (HRD) and have recently been approved by the FDA for the treatment of germline *BRCA1/2-*mutation-associated breast cancer. PARPis have also been found to be efficacious in *BRCA* wild-type (*BRCAwt*) lesions with high genomic loss of heterozygosity (LOH-high). The goal of this study was to retrospectively investigate the tumor mutations in homologous recombination (HRR) genes and the LOH score in advanced-stage breast carcinomas (BCs). Sixty-three patients were included in our study, 25% of whom had HRR gene mutations in their tumors, including 6% *BRCA1/2* and 19% non-*BRCA*-containing gene mutations. An HRR gene mutation was associated with a triple-negative phenotype. Twenty-eight percent of the patients had an LOH-high score, which, in turn, was associated with a high histological grade, a triple-negative phenotype, and a high tumor mutational burden (TMB). Among the six patients who received PARPi therapy, one had a tumor with a *PALB2* mutation other than *BRCA* and had a clinical partial response. Twenty-two percent of the LOH-low tumors had *BRCAwt*–HRR gene mutations, compared with 11% of the LOH-high tumors. Comprehensive genomic profiling revealed a subset of breast cancer patients with a *BRCAwt*–HRR gene mutation that would be missed by an LOH test. The necessity of next-generation sequencing coupled with HRR gene analysis for PARPi therapy requires further investigation in clinical trials.

## 1. Introduction

The prevalence of *BRCA1* or *BRCA2* germline pathogenic mutations is approximately 5% in patients with breast cancer [1,2]. Tumors carrying a *BRCA1* mutation are more often of a higher histological grade or of the triple-negative type (estrogen receptor (ER) negative, progesterone receptor (PR) negative, and human epidermal growth factor receptor 2 (HER2) negative) with a prominent lymphocytic infiltrate, whereas *BRCA2* tumors are more often ER positive [3,4]. Proteins encoded by the *BRCA1* and *BRCA2 (BRCA1/2)* genes are critical for homologous recombination (HRR) DNA repair [5]. In opposition, poly (adenosine diphosphate-ribose) polymerase inhibitors (PARPis) kill tumor cells that have homologous recombination repair deficiency (HRD) [6]. PARPis have shown activity in *BRCA1/2*-associated breast, ovarian, prostate, and pancreatic cancers [7,8,9]. The U.S. Food and Drug Administration (FDA) and the European Medicines Agency (EMA) approved olaparib and talazoparib for treatment of patients with germline *BRCA*-mutated (*gBRCAm*), HER2-negative, locally advanced or metastatic breast cancer [10,11,12]. However, it is not clear if the HRR gene mutation status beyond *BRCA* genes can be used as a candidate biomarker for PARPi in breast cancer. 

Previous studies have demonstrated that tumor cells with a deficiency in other HRR proteins also showed sensitivity to PARPi [13,14]. Clinical trials demonstrated that PARPi benefited a subgroup of ovarian cancer patients who had *BRCA* wild-type (*BRCAwt*) tumors with high genomic loss of heterozygosity (LOH) or identifiable mutations in other HRR genes [15,16,17]. In breast cancer, Oshi et al. established a novel *BRCA*ness score that predicted the response to PARPi regardless of *BRCA* mutation [18]. However, the predictive value of other specific HRR genes for PARPi therapy has yet to be confirmed in clinical trials. 

An algorithm integrating the mutation signatures in HRR that can both identify *BRCA1/2* germline and sporadic-mutation-associated breast cancer has been advanced [19]. However, the clinical application of those mutation signatures is untested. LOH is a measure of genomic instability and can be used as a surrogate marker of HRD [20,21]. The goal of this study was to retrospectively investigate tumor mutations in HRR genes along with LOH status and correlate these parameters with clinicopathological features of advanced-stage breast carcinomas (BCs). We believed our study would provide insight into important therapeutic decisions in advanced breast cancer patients.

## 2. Materials and Methods

### 2.1. Study Cohort and Clinical Data Collection

Patients diagnosed and treated for invasive breast carcinoma at our institution between 2019 and 2022 whose tumors underwent comprehensive next-generation sequencing (NGS) were identified through a UAB Institutional Review Board (IRB)-approved retrospective protocol (IRB-300006547). Sixty-three eligible patients had key demographics along with their sequencing analysis data collected. Patient demographic and clinical characteristics were obtained from the electronic medical records of our institution, including age at primary diagnosis, clinical stage at presentation, pathological stage, history of surgery, systemic therapy, and clinical treatment response. Patients who had a clinical disease stage of IIB or above were included in this study. 

### 2.2. Histology

All histopathology slides were reviewed independently by two pathologists and the pathologic characteristics were affirmed, including sampling site, histologic grade, histologic type, and prognostic and predictive marker status. The American Society of Clinical Oncology (ASCO)/College of American Pathologists (CAP) guideline recommendations [22,23,24] were used as references for categorizing ER, PR, and HER2 status as part of the routine pathologic evaluation. Tumors with low ER positivity (1–10%) were considered as ER positive in this study. Based on receptor status, patients were categorized as: ER/PR-positive (ER and/or PR positive); HER2-positive (HER2 positive regardless of ER and PR status); or triple-negative breast cancer (TNBC) (ER, PR, and HER2 negative). 

### 2.3. Comprehensive Genomic Profiling (CGP) by Next-Generation Sequencing 

Formalin-fixed paraffin-embedded (FFPE) tumors from 34 patients were subjected to whole-exon sequencing (WES) with enrichment of ~700 clinically relevant genes and whole transcriptome sequencing with genomic signatures, including microsatellite instability (MSI), tumor mutation burden (TMB), and HRD score (LOH). FFPE tumors from the remaining 29 patients were subjected to an FDA-approved CGP, targeting 324 key cancer-related genes as well as genomic signatures (MSI and TMB). Genomic alterations and genomic signatures were collected. In this study, pathogenic and likely pathogenic alterations were considered as carrying a pathogenic mutation; gene alterations with a variant of uncertain significance (VUS) were excluded. For LOH analysis, data from the ARIEL3 PARPi trial in patients with ovarian carcinoma proposed an LOH cutoff of 16% [17]. Therefore, for correlative analysis, 16% [17,25] was used as the cutoff for LOH in the current study.

Based on HRR gene alteration status, this cohort was divided into four groups: a *BRCA1-*mutated (*BRCA1*m) group, a *BRCA2-*mutated (*BRCA2*m) group, a *BRCA* wild-type and other-HRR-mutated (*BRCAwt*–HRRm) group, and a group with both *BRCA* and HRR wild-type (*BRCAwt–*HRRwt) [26]. Patients whose tumor had both a *BRCA* mutation (*BRCAm*) and a mutation in any other HRR-related gene were included in the *BRCA*m group. The HRR genes were as described in Hodgson et al.’s study [26]. The HRR genes *ATM*, *BRIP1*, *PALB2*, *BARD1*, *CHEK1*, *CHEK2*, *RAD51B*, *RAD51C*, *RAD51D*, *RAD54L*, and *FANCA* were included in both CGPs, whereas *EMSY*, *FANCD2*, *MRE11*, and *RAD50* were included only in the WES-based CGP.

### 2.4. Treatment Effect of PARPi

In this study, the effect of clinical treatment was assessed by comparing radiological studies before and after PARPi therapy after 3 months or longer and was graded as a complete response (CR), partial response (PR), stable disease (SD), or progressive disease (PD). Any new detectable lesions marked the patient as having a PD. Complete response was defined as the disappearance of all lesions; partial response was defined as a ≥50% decrease in tumor size; stable disease was defined as the tumor falling between a <50% decrease and a <25% increase; progressive disease was defined as a ≥25% increase in tumor size. 

### 2.5. Statistical Analysis

Differences between the groups in categorical variables were calculated with the Fisher exact test. Spearman correlation coefficient analysis was conducted using two continuous or ordinal variables. A *t*-test was used to compare the means of two groups. Statistical significance was established at *p* < 0.05. 

## 3. Results

### 3.1. Clinicopathological Features

Sixty-three patients with locally advanced or metastatic BC were included in the study; their clinicopathological characteristics are summarized in Table 1. The patients’ mean age was 58 years (range, 26–86 years). Sixty (95%) patients presented with clinical stage 3 or 4 disease. Twenty-six (42%) patients had a pathological T3 or T4 disease. Forty (63%) patients had regional lymph node metastasis (pN1-pN3). Thirty-eight (60%) tumors were Nottingham histological grade 3. Thirty-four (54%) tumors were in the ER/PR-positive group; 8 (13%) in the HER2 group; and 21 (33%) were of the TNBC. Specimens were collected from breast (18, 29%), liver (18, 29%), lymph nodes (8, 13%), skin (4, 6%), brain (3, 5%), soft tissue (3, 5%), bone (3, 5%), fallopian tubes (1, 2%), cervix (1, 2%), pleura (1, 2%), lung (1, 2%), bowel (1, 2%), and mediastinum (1, 2%).

### 3.2. Association between Pathologic Factors and HRD

**Figure 1:**
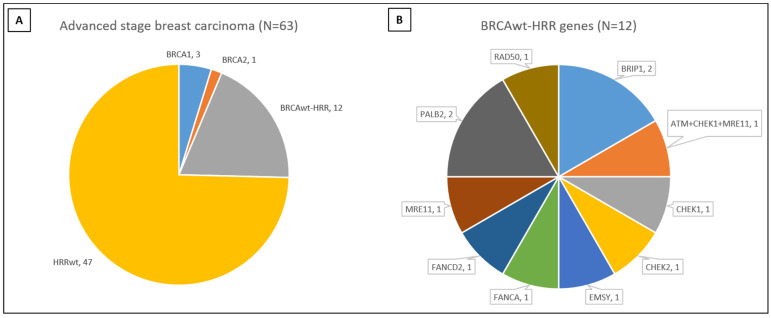
Distribution of HRR gene mutations. (**A**) Overall, 16 of 63 (25%) carcinomas had mutations in HRR genes. Among the 16 carcinomas, 4 (25%) had *BRCA1/2* mutations and 12 had other HRR gene mutations. (**B**) Distribution of other HRR gene mutations. *BRCA1/2* with additional HRR mutations were included in *BRCA1/2* carcinomas.

Of the 63 tumors, 16 (25%) had HRR mutations, including 3 (5%) *BRCA1*, 1 (1%) *BRCA2*, and 12 (19%) that had other HRR genes implicated: *ATM, BRIP1, CHEK1, CHEK2, EMSY, FANCA, FANCD2, MRE11, PALB2, RAD50,* and *RAD51C* (Supplementary material File S1). Three tumors had more than one HRR gene mutation: one in *BRCA1* and *FANCA*; one in *BRCA2* and *RAD51C*; and one in *ATM, CHEK1,* and *MRE11* (Figure 1). HRR mutations were associated with N3-stage disease and HER2 negativity. Of the 63 tumors, 55 had an available TMB score and no significant association with LOH (*p* = 0.2538). LOH and TMB data were available for 32 tumors. LOH-high was associated with T4- or N3-stage disease, Nottingham grade 3, ER negativity, and the TNBC (Table 2). The LOH score was positively associated with TMB (*p* = 0.0234).

### 3.3. Association between LOH and HRR Genes

**Figure 2:**
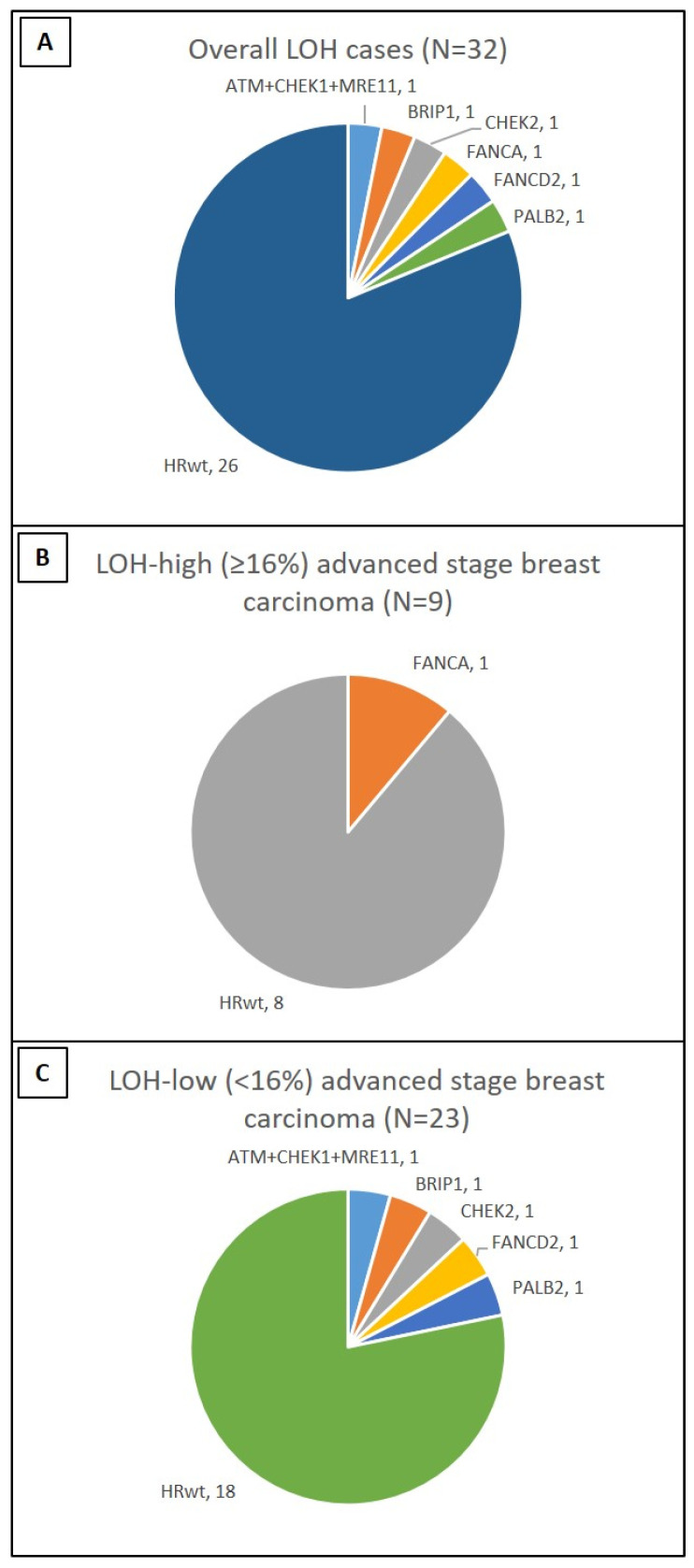
Distribution of HRR gene mutations in carcinomas with an available LOH score. (**A**) There were 6 of 32 (20%) carcinomas with HRR gene mutations. (**B**) By using a 16% cutoff, an LOH-high score was identified in only 1 of 6 (17%) carcinomas with HRR gene mutations. (**C**) An LOH-low score was identified in 5 of 6 (83%) carcinomas with HRR gene mutations.

Of the 32 tumors with available LOH data, none had a *BRCA* mutation and 6 (19%) had *BRCAwt*–HRR mutations, involving *ATM, BRIP1, CHEK1, CHEK2, FANCA, FANCD2, MRE11,* and *PALB2* genes. One tumor had *ATM*, *CHEK1,* and *MRE11* mutations (Figure 2A). Of the 32 tumors, 9 (28%) were LOH-high (Figure 2B). Of the 23 LOH-low tumors, 5 (22%) had *BRCAwt–*HRR mutations (Figure 2C).

### 3.4. Clinical Response of PARPi Therapy 

Out of 63 patients, 6 (9%) received PARPi therapy in the context of a clinical trial; the clinicopathological characteristics of these are summarized in Table 3. Of the primary tumors from these six patients, two were histological grade 2 and four were grade 3; two were ER/PR-positive, one was HER2-positive, and three were of the TNBC type. Two patients had a tumor with a *BRCA1* mutation, one had *BRCA1* and *FANCA* mutations, one had a *PALB2* mutation, one had a *BRCA1* VUS, and one had a *BRCA2* VUS. All six patients received surgery and systemic chemoradiation therapy. Based on the short period of follow-up after the initiation of PARPi treatment, two patients had progressive disease, one had a partial response, one had stable disease, one was disease free, and one discontinued the treatment due to intolerable side effects. 

**Figure 3:**
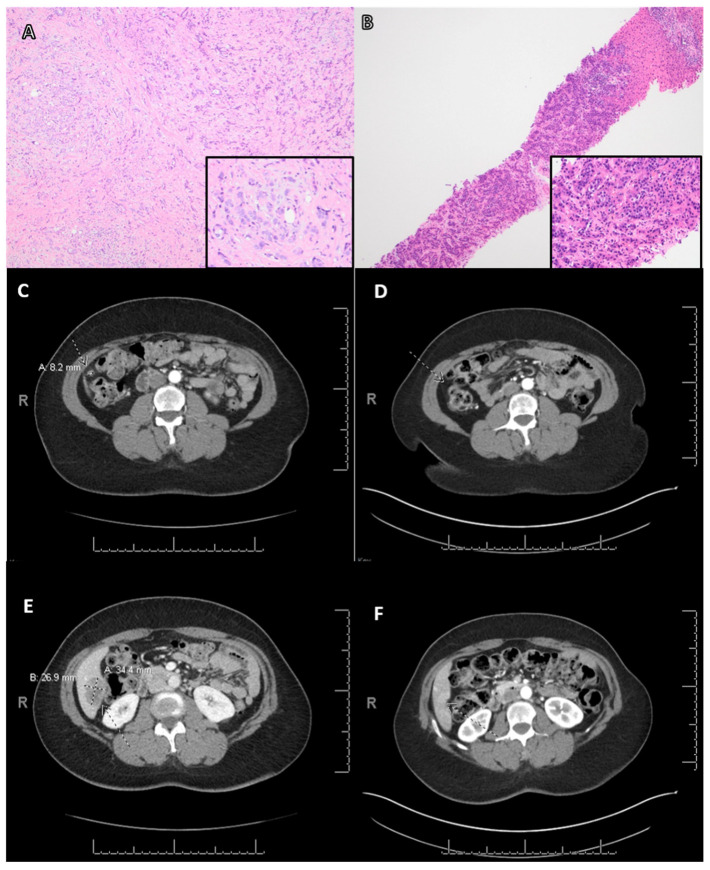
Clinical response of PARPi in a patient with advanced-stage breast carcinoma carrying a *PALB2* mutation. Biopsy of orbital (**A**) and liver (**B**) lesions revealed metastatic carcinoma of breast origin. (Hematoxylin and eosin stain, original magnification: A and B: ×200). (**C**) Computed tomography (CT) showed a baseline peritoneal lesion measuring 0.8 cm. (**D**) At seven months follow-up, a CT showed resolution of this peritoneal lesion. (**E**) CT revealed a baseline liver lesion measured 2.7 cm. (**F**) At seven months follow-up, a CT showed this lesion decreased in size to 0.7 cm.

Three of the six patients are worth special mention. The first patient (case No. 1, Table 3) was a 52-year-old woman with a 1.7 cm ER-positive/HER2-negative, histological grade 2 invasive carcinoma of no special type (IC-NST), who underwent breast-conserving surgery, adjuvant docetaxel, and cyclophosphamide and radiation therapy. During 61 months of follow-up after her initial diagnosis, she developed orbital, liver, and peritoneal metastasis. Biopsies of the orbital and liver lesions revealed metastatic ER-positive/HER2-negative BC. An NGS assay performed on the orbital tumor showed a *PALB2* mutation, and she also had a known germline *PALB2* mutation. Thus, the patient was started on a clinical trial with PARPi. A follow-up CT scan performed 3 months after initiation of treatment showed a stable orbital lesion. On a 7-month follow-up, the CT showed a decreased size in both the liver and peritoneal lesions without new lesions being present. Figure 3 shows the pathologic and radiologic findings of this patient.

**Figure 4:**
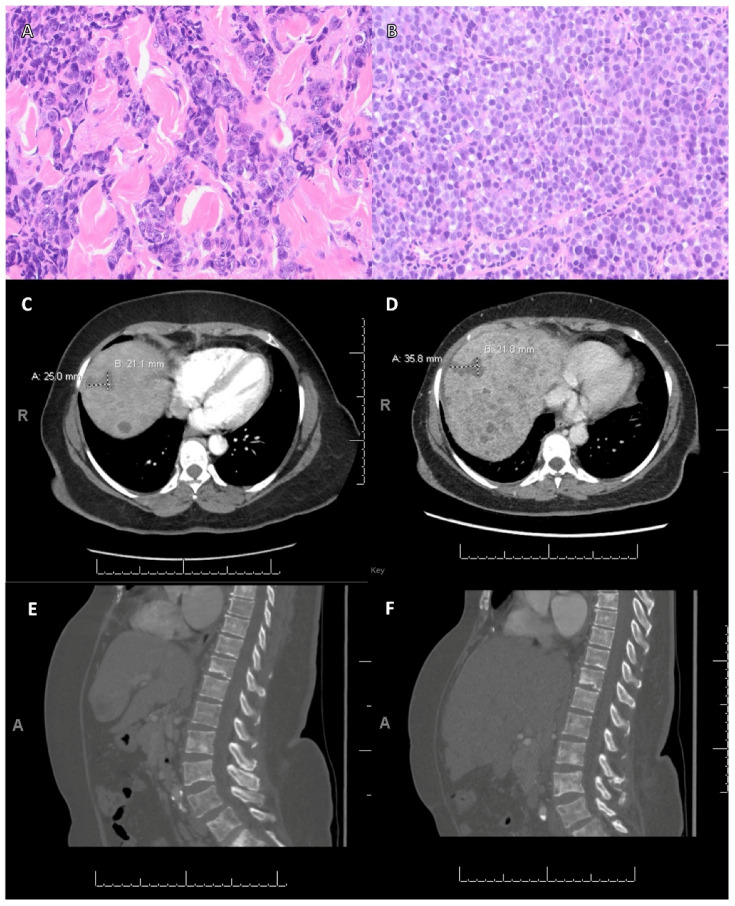
The clinical response of PARPi in a patient with advanced-stage breast carcinoma carrying *BRCA1* and *FANCA* mutations. (**A**) Biopsy specimen of a 1.8 cm breast mass showed invasive ductal carcinoma. (**B**) Biopsy of axillary lymph node revealed metastatic carcinoma of breast. (Hematoxylin and eosin stain, original magnification: A and B. ×200.) (**C**) Computed tomography (CT) showed a baseline liver lesion measured 2.5 cm. (**D**) Three-month follow-up CT showed this lesion increased in size to 3.6 cm. (**E**) CT showed extensive scattered mixed lytic and sclerotic bone lesions throughout the spine and pelvis before PARPi therapy. (**F**) Three-month follow-up CT showed extensive osseous metastases with no significant changes.

The second patient (case No. 4, Table 3) was a 60-year-old woman with histological grade 3, ER-positive/HER2-negative invasive carcinoma of no special type (IC-NST) involving bilateral breasts and axillary lymph nodes, after bilateral total mastectomy and adjuvant therapy with doxorubicin, cyclophosphamide, paclitaxel, letrozole, and tamoxifen. Seven years later, she developed liver and bone metastasis. An NGS assay was performed on a liver lesion sample and showed pathogenic mutations in *BRCA1* and *FANCA*. She was enrolled in a clinical trial and started on olaparib. A follow-up computed tomography (CT) scan performed 3 months after the initiation of PARPi demonstrated an enlarged liver with an increase in size and number of hepatic metastases but unchanged extensive osseous metastases. Figure 4 shows the pathologic and radiologic findings of this patient.

**Figure 5:**
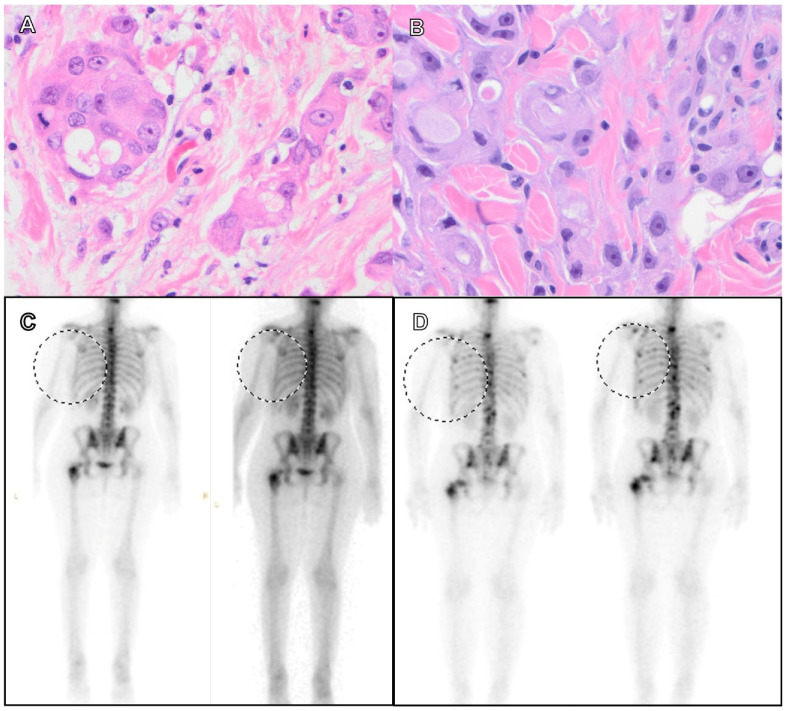
Clinical response of PARPi in a patient with advanced-stage breast carcinoma carrying a *BRCA* VUS. (**A**) Breast resection showed multifocal invasive ductal carcinomas. (**B**) Biopsy of a neck lesion demonstrated adenocarcinoma in the dermis of breast origin. (Hematoxylin and eosin stain, original magnification: A and B. ×400.) (**C**) Nuclear medicine (NM) bone scan showed multifocal osseous metastatic disease, including the anterior right fifth and sixth ribs before PARPi therapy. (**D**) At the four-month follow-up, a bone scan showed multifocal new osseous metastases and new patchy uptake in bilateral ribs.

The third patient (case No. 5, Table 3) was a 46-year-old woman with clinical stage T4, histological grade 3, TNBC who underwent neoadjuvant and adjuvant chemotherapy with carboplatin, paclitaxel, and capecitabine following a total mastectomy. Twenty-nine months later, she developed metastatic disease in her contralateral breast and bone. An NGS assay performed in the metastatic breast tumor showed *AKT, TP53,* and *NF1* mutations and a *BRCA1* VUS. She also had a known germline *NF1* mutation. She started olaparib as a first-line therapy within a short period of time after the neoadjuvant chemotherapy and total mastectomy. Four months later, the follow-up nuclear medicine (NM) bone scan showed progressive bone disease. Thus, she was switched to atezolizumab and paclitaxel, and three months later, she developed brain metastasis. Figure 5 shows the pathologic and radiologic findings of this patient.

## 4. Discussion

Poly (adenosine diphosphate-ribose) polymerase inhibitors (PARPis) target tumors with a homologous recombination deficiency (HRD). The EMBRACA phase 3 trial showed that the single-agent talazoparib significantly benefited patients with advanced breast cancer and a germline *BRCA1/2* mutation over standard chemotherapy [11]. Recently, the OlympiA phase 3 trial showed that patients with high-risk, HER2-negative early breast cancer who have germline *BRCA1* or *BRCA2* pathogenic or likely pathogenic alterations benefited from adjuvant olaparib [27]. Whether there are other biomarkers to select breast cancer patients for PARPi therapy is not clear. Our study investigated *BRCA1/2* and other HRR gene mutations and LOH status in a cohort of locally advanced or metastatic breast carcinomas. 

It has been demonstrated that breast cancers with *BRCA1* mutations are more likely to be of high histological grade or triple-negative compared with tumors with a *BRCA2* mutation [3]. In our study, 16 of 63 (25%) tumors had pathogenic or likely pathogenic mutations in the HRR genes included in the tested panel: *BRCA1*, *BRCA2*, *ATM, BRIP1, CHEK1, CHEK2, EMSY, FANCA, FANCD2, MRE11, PALB2, RAD50,* and *RAD51C.* The tumors with those HRR mutations (compared with HRRwt) were more frequently HER2 negative (*p* < 0.05). Among the 16 tumors with HRR gene mutations, 3 (19%) had a *BRCA1* mutation and 2 of them were of the TNBC type (2/3). Among the remaining 13 tumors (including those carrying *BRCA2* and *BRCAwt*–HRRm), 5 were of the TNBC type (5/13). Although our study demonstrated a positive association between having HRR gene mutations and HER2 negativity, any associations between each subgroup and receptor status were too small to be statistically meaningful and require a larger cohort. 

Another PARPi phase 2 trial showed a 41% overall response rate in patients with *BRCA*-deficient advanced breast cancer [7]. A subgroup of these patients carrying a *BRCA* mutation did not respond to therapy. Multiple mechanisms of resistance to PARPi have been proposed [28,29,30], such as drug-target-related resistance, restoration of homologous recombination, and restoration of replication fork stability. Clinical confirmation of those identified mechanisms is necessary. In our study, three patients whose tumor had a *BRCA1* mutation received PARPi therapy. One patient had stable disease (case No. 3, Table 3) and one was free of disease for 40 months (case No. 2) after initiation of PARPi as adjuvant therapy. The third patient (case No. 4) carrying a *BRCA1* and an *FANCA* mutation in the tumor had progressive disease after initiation of PARPi. It has been demonstrated that *MRE11*-mediated fork degradation is suppressed by *FANCD2* and that *FANCD2* overexpression leads to PARPi resistance [30]. An *FANCA* mutation involved in the Fanconi anemia pathway might have partially contributed to PARPi resistance in this patient. 

Several studies have also shown that PARPi benefited patients lacking *BRCA* mutations [17,31,32]. A phase II study of olaparib monotherapy demonstrated antitumor activity in patients with germline *PALB2* mutations [33]. In our study, 19% (12/63) of the breast cancers had *BRCAwt*–HRR mutations compared with 6% (4/63) that had *BRCA1/2* mutations. Tumors from two patients had *PALB2* mutations but lacked *BRCA* mutations. One of the two patients received PARPi therapy (case No. 1, Table 3) and had a partial response to the treatment. PARPi showed anti-tumor activity in this patient with a *PALB2* mutation, further supporting previous studies. Detecting loss-of-function mutations in HRR genes other than *BRCA1/2* may recognize an additional small subgroup of patients with breast cancer susceptible to PARPi therapy. 

The frequency of a variant of uncertain significance (VUS) of *BRCA1/2* in breast cancer varies among different studies; a recent study reported a rate of 9% mostly in non-*BRCA1-* or *BRCA2-*carrying tumors [34]. Although the study showed that the survival outcome of *BRCA* VUS carriers is comparable to *BRCAwt* patients in ovarian cancer [35], the predictive value for PARPi therapy in *BRCA* VUS remains unclear. Among our 63 patients, 7 (11%) had tumors with HRR-VUS, including 3 (5%) associated with *BRCA1*; 3 (5%) with *BRCA2*; and 2 (3%) were associated with other HRR genes. One patient had a VUS in both *BRCA2* and *CHEK2* genes in her tumor. Data are shown in Supplementary Figure S1. Two of the seven patients received PARPi therapy, one had progressive disease (case No. 5, Table 3), and another had to discontinue treatment due to PARPi-induced pneumonitis (case No. 6, Table 3). The first patient had a known germline *NF1* mutation and had *AKT, TP53,* and *NF1* mutations as well as a *BRCA1* VUS in the metastatic tumor. An *NF1* mutation in breast cancer has been found to be associated with ER/PR negativity, HER2 amplification, and worse survival [36]. Our patient had a germline *NF1* mutation, and her ER/PR-negative HER2-positive breast cancer showed a poor response to systemic treatment involving chemotherapy, an immune checkpoint inhibitor, and PARPi. In our cohort, tumors appear to harbor VUS in *BRCA1/2* more often than in other HRR genes, in agreement with a previous study [37]. This particular case did not show any predictive value of *BRCA* VUS for PARPi therapy. 

Loss of heterozygosity (LOH) is a genomic test (via an NGS assay) to determine the percentage of HRD. Such an LOH test has not been validated in breast cancer. A standardized threshold to define LOH-high versus LOH-low has not been fully developed. The ARIEL3 trial showed that patients with a *BRCAwt* and high LOH (≥16%) recurrent ovarian carcinoma benefited from rucaparib treatment [17]. In our study, tumors from 32 patients had an LOH score available. At a cutoff of 16%, 28% (9/32) of the tumors were LOH-high, which was associated with a pT4- or pN3-stage disease, Nottingham histological grade 3, triple-negative phenotype, and ER negativity. The PrECOG0105 phase 2 trial showed that the LOH test identified patients with triple-negative breast cancer with a high LOH (≥10%) score, and lacking *BRCA1/2* mutation, who achieved a favorable pathologic response to iniparib in combination with chemotherapy. In our study, using a cutoff of 10%, 66% (21/32) of tumors were LOH-high, which was associated with a pT4- or pN3-stage disease, triple-negative phenotype, and ER negativity (Supplementary Table S1). We believe this study is the first clinical study to report the positive association between an LOH-high score and unfavorable pathological features. However, the value of this test as a predictive and/or prognostic marker for PARPi requires further study. 

Genomic alterations in *BRCA1/2* have been highly associated with HRD in many cancers [25,38]. Sokol et al. showed that a *BRCA1/2* alteration was consistently associated with LOH-high in many cancer types, including breast cancer, and the magnitude was variable for each cancer type. In breast cancer, although more than 75% of cases with *BRCA1/2* alterations were LOH-high, >25% of *BRCA1/2* wild-type (*BRCA1/2wt*) cases were LOH-high [25]. In our study, 32 cases with an available LOH score were all *BRCA1/2wt,* and 28% (9/32) were LOH-high. The cases included in the current cohort were not a random selection, at risk for sampling bias. Multiple clinical and preclinical studies have established that non-*BRCA1/2* HRR genes, such as *ATM, BRIP1, CHEK1, CHEK2, CDK12, PALB2, RAD51C,* and *RAD51D*, also confer sensitivity to PARPi [15,33]. Interestingly, in our study, many of those gene mutations, such as *ATM, BRIP1, CHEK1, CHEK2,* and *PALB2,* were identified in LOH-low tumors. Upon using a <10% cutoff, *PALB2, ATM,* and *MRE11* mutations were identified in LOH-low tumors (Supplementary Figure S2). Although these individual HRR genes have not been validated as predictive markers for PARPi at present, a single LOH test failed to recognize this subset of tumors with HRD. Although an LOH test can recognize a subset of breast cancers with a *BRCA1/2* mutation for potential PARPi treatment, comprehensive genomic profiling may be required to target a larger patient population. 

## 5. Conclusions

Pathogenic mutations in HRR genes were present in 25% of 63 BCs and were associated with triple negativity. Of 63 patients, 19% carried a non-*BRCA* HRR gene mutation in their tumors, and PARPi was effective against some of these tumors. An LOH-high score showed a positive association with a high histological grade and triple negativity, and only identified a subset of BCs with a non-*BRCA* gene mutation. We hypothesize that next-generation sequencing with full HRD gene analysis should be considered when PARPi treatment is contemplated in advanced breast cancer. However, this hypothesis requires further confirmation in clinical trials of PARPi with full genetic status characterization. 

## Figures and Tables

**Table 1:** Clinicopathological features of patients with advanced-stage breast carcinoma.

Characteristic	All Patients (n = 63)	
	Number	%
**Clinical stage**		
I and IIA	0	0
IIB	3	5
III	4	6
IV	56	89
**T classification**		
pT1	10	16
pT2	22	35
pT3	13	21
pT4	13	21
Not available	5	8
**N classification**		
pN0	18	29
pN1	28	44
pN2	4	6
pN3	8	13
Not available	5	8
**M classification**		
pM0	7	11
pM1	56	89
**Nottingham histological grade**		
1	1	2
2	24	38
3	38	60
**Histological subtype**		
Ductal	56	89
Lobular	7	11
**Estrogen Receptor status**		
Positive	37	59
Low positive	2	3
Negative	24	38
**Progesterone Receptor status**		
Positive	32	51
Negative	31	49
**HER2 status**		
Positive	8	13
Negative	55	87
**Triple-negative type**		
Yes	21	33
No	42	67
**History of chemoradiation therapy**		
Yes	55	87
No	8	13
**History of hormonal therapy**		
Yes	41	65
No	22	35
**History of immune checkpoint inhibitor therapy**		
Yes	11	17
No	52	83
**PARPi therapy**		
Yes	6	10
No	57	90

**Table 2:** Association between HRR and pathologic features.

Factors		HRR (n = 63)				LOH (16%Cutoff) (n = 32) **		
		HRRmt	HRRwt	*p* Value		LOH (≥16%)	LOH (<16%)	*p* Value
Pathologic T classification *	T1T2T3 (n = 45)	13 (29%)	32 (71%)	0.0169	T1T2T3 (n = 23)	6 (26%)	17 (74%)	**0.0114**
	T4 (n = 13)	2 (15%)	11 (85%)		T4 (n = 7)	3 (43%)	4 (57%)	
Pathologic N classification *	N0N1N2 (n = 50)	10 (20%)	40 (80%)	**<0.00001**	N0N1N2 (n = 26)	7 (27%)	19 (73%)	**0.0008**
	N3 (n = 8)	5 (63%)	3 (38%)		N3 (n = 4)	2 (50%)	2 (50%)	
Nottingham grade	1 and 2 (n = 25)	6 (24%)	19 (76%)	0.7440	1 and 2 (n = 11)	2 (18%)	9 (82%)	**0.01495**
	3 (n = 38)	10 (26%)	28 (74%)		3 (n = 21)	7 (33%)	14 (67%)	
Triple negativity	Yes (n = 21)	7 (33%)	14 (67%)	0.0560	Yes (n = 7)	4 (57%)	3 (43%)	**<0.00001**
	No (n = 42)	9 (21%)	33 (79%)		No (n = 25)	5 (20%)	20 (80%)	
ER	Pos. (n = 39)	8 (21%)	31 (79%)	0.0560	Pos. (n = 22)	4 (18%)	18 (82%)	**<0.00001**
	Neg. (n = 24)	8 (33%)	16 (67%)		Neg. (n = 10)	5 (50%)	5 (50%)	
PR	Pos. (n = 32)	7 (22%)	25 (78%)	0.2561	Pos. (n = 18)	5 (28%)	13 (72%)	0.8755
	Neg. (n = 31)	9 (29%)	22 (71%)		Neg. (n = 14)	4 (29%)	10 (71%)	
HER2	Pos. (n = 8)	1 (13%)	7 (88%)	**0.0121**	Pos. (n = 6)	2 (33%)	4 (67%)	0.3545
	Neg. (n = 55)	15 (27%)	40 (73%)		Neg. (n = 26)	7 (27%)	19 (73%)	

HRR: Homologous recombination repair, LOH: Loss of heterozygosity, Pos.: Positive, Neg.: Negative. * Fifty-eight patients had an available pathologic T and N classification. ** Thirty-two tumors had an available LOH score from whole-exon sequencing (WES).

**Table 3:** Clinicopathological characters of patients with PARPi therapy.

Case	Age	Primary Tumor ^a^					Lymph Node Status	History of Treatment				NGS ^g^		LOH (%)	TMB (Muts/Mb)	PARPi ^d^	Follow-Up ^e^ (Month)	Clinical Response ^f^
		Diagnosis	NG	ER	PR	HER2		Surgery ^b^	Systemic Therapy ^c^	Radiation	Method	HRR Gens	Non-HRR Gens					
1	46	IC-NST	2	Pos	Pos	Neg	Neg	PM	Docet Cytoxan Tamox	Yes	IVD	*PALB2* p.Y1108fs*6 *PALB2* p.K480fs*6	*Rad21* Amplified *RARA* p.M284I	N/A	3	O	7	PR
2	59	IC-NST	3	Neg	Neg	Neg	Neg	TM	Docet Cytoxan Doxor Pembro	Yes	IVD	*BRCA1* p.V757fs*8	*PTEN* loss *MYC* amplified *CDKN2A/B* loss *EP300* truncation intron 27 *FAS* loss *GATA6* amplified *LRP1B* p.R441* MCL1 amplified *NUP93* p.R709T *TP53* p.L257P	N/A	5	O	40	DF
3	20	IC-NST	3	Neg	Neg	Neg	Pos	TM	Doxor Cytoxan Carbo Taxol	Yes	IVD	*BRCA1* p.R1751	*CTNNA1* p.E686fs*39 *RB1* splice site 2063–2106+20del64 *TP53* p.R175H	N/A	1	O	6	SD
4	60	IC-NST	3	Pos	Pos	Neg	Pos	TM	Doxor Carbo Tamox	Yes	IVD	*BRCA1* p.C903fs*97 *FANCA* p.E63*	*FGF12* amplified *MYC* amplified *PIK3CA* p.P104del *SF3B1* p.K700E *SOX2* amplified	N/A	6	O	3	PD
5	47	IC-NST	3	Neg	Neg	Neg	Pos	TM	Atezo Nab-p	Yes	IVD	*BRCA1* p.K1183R VUS	*AKT*1 p.E17K *TP53* p.C141Y *NF1* p.E1334*	N/A	N/A	O	4	PD
6	59	IC-NST	2	Neg	Neg	Pos	Pos	TM	Docet Herce Pertu	Yes	WES	*BRCA2* p.V188M VUS *CHEK2* p.Y139H VUS	*AKT1* p.E17K *ERBB2* amplified *SPEN* c.1624–1635+1del13 *TP53* p.R248Q	6	3	O	1	Discontinued

^a^ IC-NST: Invasive carcinoma of no special type, NG: Nottingham grade, Pos: Positive, Neg: Negative; ^b^ PM: Partial mastectomy, TM: Total mastectomy; ^c^ NACT: Neoadjuvant chemotherapy, ACT: Adjuvant chemotherapy, N/A: Not applicable, Docet: Docetaxel, Cytoxan: cyclophosphamide, Tamox: Tamoxifen, Atezo: Atezolizumab, Nab-p: Nab-paclitaxel, Herce: Herceptin, Pertu: Pertuzumab, Pembro: Pembrolizumab, Doxor: Doxorubicin; ^d^ Olaparib; ^e^ FU: Follow-up after initiation of PARPi; ^f^ SD: Stable disease, PD: Progressive disease, DF: Disease free; ^g^ VUS: variant of unknown significance, IVD: in vitro diagnostic test, WES: Whole-exon sequencing.

## Data Availability

Data is contained within the article; raw data are available upon request from the corresponding author.

## Supplementary Materials

The following supporting information can be downloaded at: https://www.mdpi.com/article/10.3390/cancers15092524/s1, Figure S1. Distribution of HRR-VUS gene mutations. Figure S2. By using a 10% cutoff, a LOH-high score was identified in 4 of 6 (67%) carcinomas with HRR gene mutations. Table S1. Association between LOH (10% cutoff) and pathologic features. File S1: Molecular profiling of the 63 tumors.
