# Supplementary material for: Mutations in Homologous Recombination Genes and Loss of Heterozygosity Status in Advanced-Stage Breast Carcinoma
Source: Cancers (Basel). 2023 Apr 28;15(9):2524. doi: 10.3390/cancers15092524 (PMC10177458; doi:10.3390/cancers15092524)
Supplement: Supplementary file 1 [file cancers-15-02524-s001.zip › cancers-2330202-Supplementary.pdf]

Supplementary Materials:

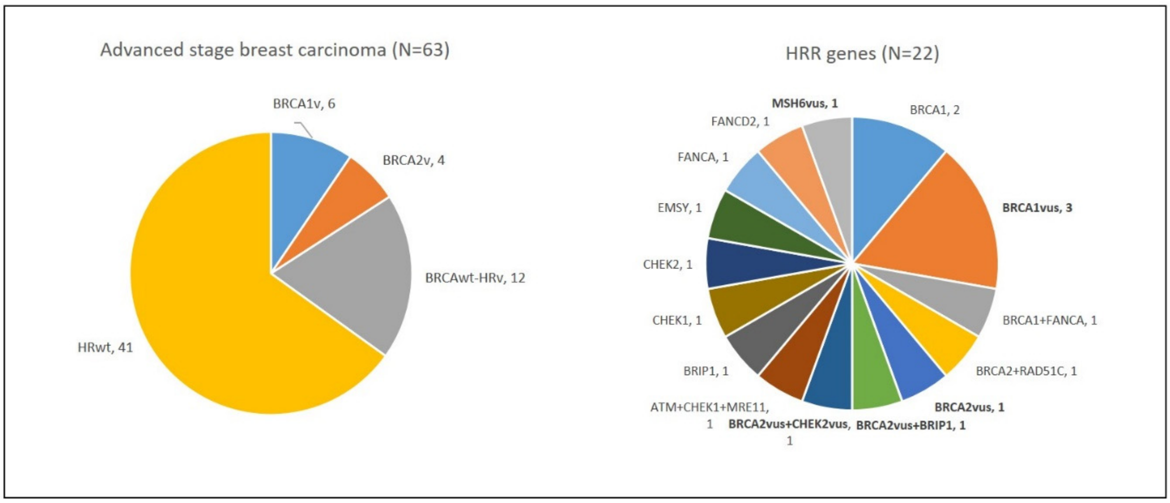

Figure S1. Distribution of HRR-VUS gene mutations.

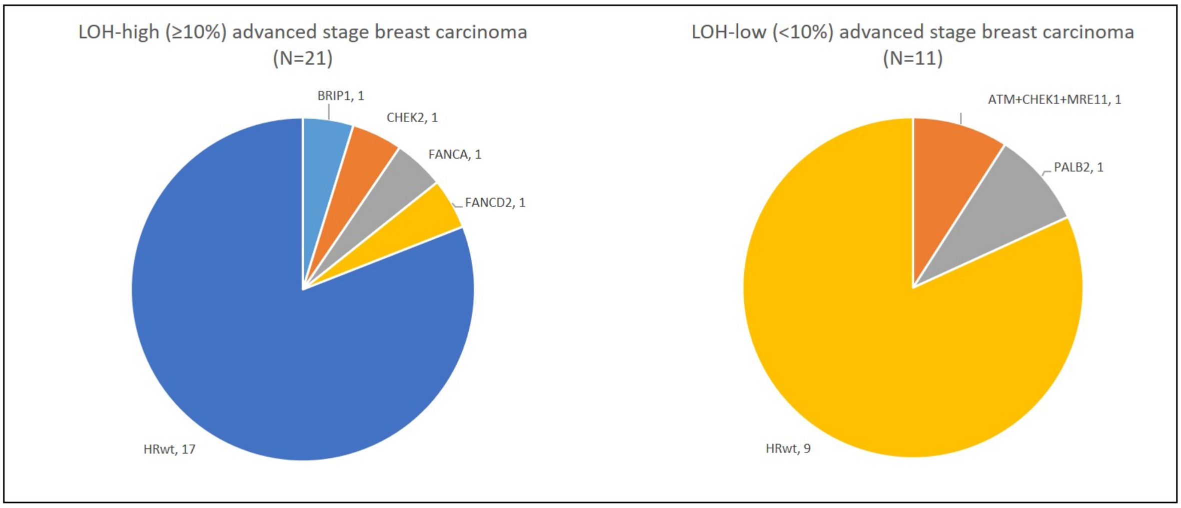

Figure S2. By using a 10% cutoff, a LOH-high score was identified in 4 of 6 (67%) carcinomas with HRR gene mutations.

Table S1. Association between LOH (10% cutoff) and pathologic features.

Factors		LOH (10%cutoff)		
		LOH (≥10%)	LOH (<10%)	P value
Pathologic T classification	T1T2T3 (n=23)	15(65%)	8(35%)	**0.0006**
	T4 (n=7)	6(86%)	1(14%)	
Pathologic N classification	N0N1N2 (n=26)	17(63%)	9(35%)	**< 0.00001**
	N3 (n=4)	4(100%)	0(0%)	
Nottingham grade	1 and 2 (n=11)	7(64%)	4(36%)	0.6554
	3 (n=21)	14(67%)	7(33%)	
Triple negativity	Yes (n=7)	7(100%)	0(0%)	**< 0.00001**
	No (n=25)	14(40%)	11(60%)	
ER	Pos. (n=22)	12(55%)	10(45%)	**< 0.00001**
	Neg. (n=10)	9(90%)	1(10%)	
PR	Pos. (n=18)	11(61%)	7(39%)	0.1356
	Neg. (n=14)	10(71%)	4(29%)	
HER2	Pos. (n=6)	4(67%)	2(33%)	0.7653
	Neg. (n=26)	17(65%)	9(35%)	

LOH Loss of heterozygosity
